# Cardiac thrombus and stroke in a child with *Mycoplasma pneumoniae* pneumonia: A case report

**DOI:** 10.1097/MD.0000000000024297

**Published:** 2021-02-05

**Authors:** Yefeng Wang, Yunbin Xiao, Xicheng Deng, Ningan Xu, Zhi Chen

**Affiliations:** a Department of Cardiology, Hunan Children's Hospital; b Department of Cardiothoracic Surgery, Hunan Children's Hospital; c Department of Health Care Center, Hunan Children's Hospital, Changsha, Hunan, China.

**Keywords:** cardiac thrombus, case report, *Mycoplasma pneumonia*, stroke

## Abstract

**Rationale:**

Cardiac thrombus and stroke are rare complications in *Mycoplasma pneumoniae* infection, which is a common cause of community-acquired pneumonia in children. Early detection and prevention of thrombus in children with *M pneumoniae* pneumonia is relatively difficult.

**Patient concerns:**

A 5-year-old boy with severe *M pneumoniae* pneumonia was referred to our center. During the treatment with sufficient antibiotics, an echocardiography surprisingly revealed a thrombus in the left atrium, with significant changes in D-dimer level and anti-phospholipid antibodies. At day 12 after admission, the patient showed impaired consciousness, aphasia, and reduced limb muscle power. Magnetic resonance angiography (MRA) showed right middle cerebral artery infarction.

**Diagnoses:**

Cardiac thrombus and stroke associated with *M pneumoniae* pneumonia.

**Interventions:**

He was started on aggressive antibiotic therapy and urokinase thrombolytic therapy for 24 hours, continued with low molecular heparin calcium and aspirin along with rehabilitation training.

**Outcomes:**

On follow up, the D-dimer decreased slowly and echocardiograms showed a steadily decreasing size of thrombus with eventual disappearance at day 22 after admission. His left limb muscle power was improved after rehabilitation for 2 months.

**Lessons:**

Early diagnosis and treatment with multiple modalities maybe useful for improving prognosis of cardiac thrombus and stroke in *M pneumoniae* pneumonia. Changes in D-dimer level and anti-phospholipid antibodies should be routinely monitored in severe *M pneumoniae* pneumonia.

## 1 Introduction

*Mycoplasma pneumoniae* is a common cause of community acquired pneumonia in children and *M pneumoniae* infection accounts for approximately 20% of pediatric pneumonia patients requiring hospitalization. It has been known to cause various kinds of extrapulmonary manifestations including vasculitis, pancreatitis, myocarditis and central nervous system sequelae. This is the first case of 1 child with both cardiac thrombus and stroke associated with *M pneumoniae* pneumonia. The related published reports on this topic have been reviewed to discuss the possible underlying mechanisms.

The ethics committee of Hunan Children's Hospital approved this study as a case report for retrospective analysis. Informed written consent was obtained from the patient parents for publication of this case report and accompanying images. We anonymized all information before analysis.

## 2 Case presentation

**Figure 1:**
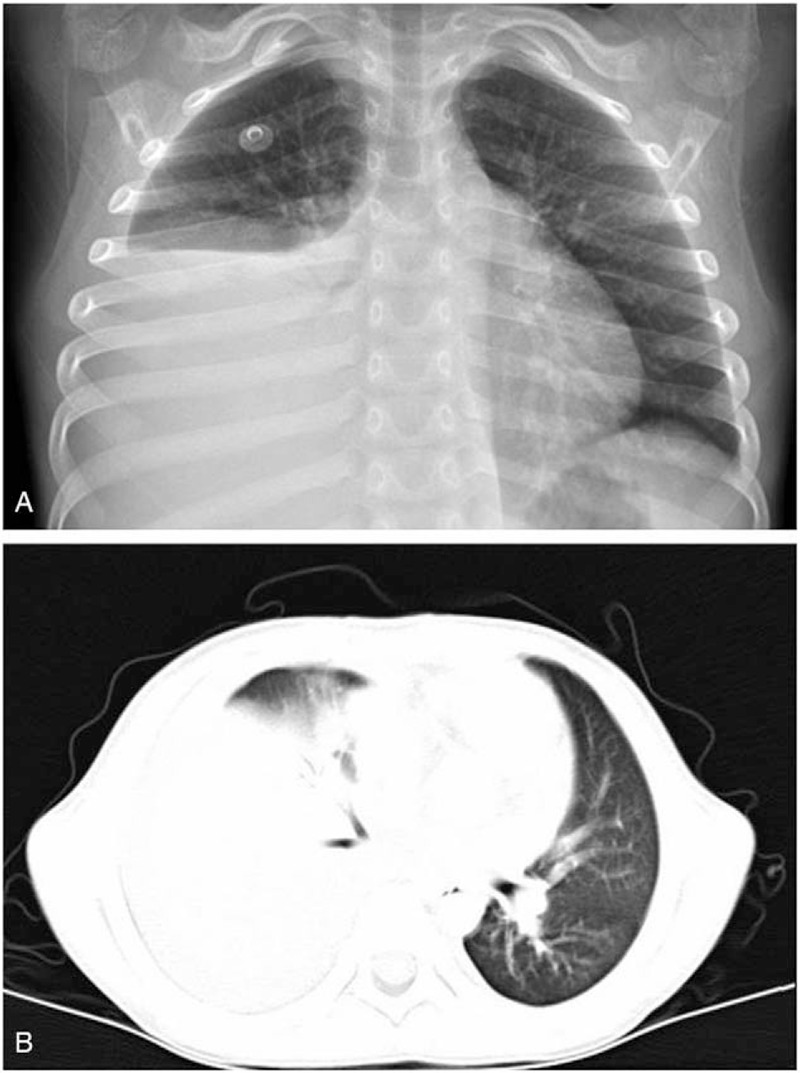
Chest X-ray and CT on admission. Chest X-ray and CT revealed a consolidation in the right lower lobe and massive pleural effusion. CT = computed tomography.

A 5-year-old boy was admitted at a local hospital because of persistent fever and dry cough for 9 days. He was diagnosed with mycoplasma pneumonia and treated with amoxicillin-clavulanic acid and azithromycin. Five days after the initial treatment, chest X-ray revealed a consolidation in the right lower lobe and massive pleural effusion (Fig. 1a). The patient was referred to our center for detailed assessment and treatment. His medical and family history was unremarkable, with no previous contact with infectious disease. He received all vaccinations as scheduled.

On admission, his body temperature was 39.4°C and the oxygen saturation was 96%with no signs of tachypnea or dyspnea. The vocal fremitus had diminished in lower right lung, where was dull on percussion. Neurological examination was unremarkable.

**Figure 2:**
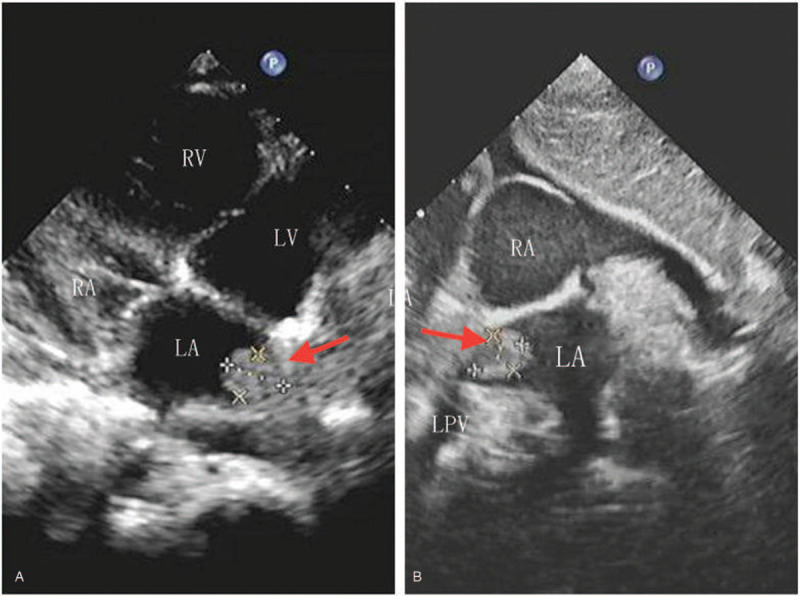
Echocardiography. The echocardiography revealed the thrombus attached to the lateral wall of the left atrium extending into the right lower pulmonary vein. LA = left atrium, LV = left ventricular, RA = right atrium, RLPV = right lower pulmonary vein, RV = right ventricular.

Laboratory testing revealed white cell count of 12400/μl and platelet count of 516000/μl, with a differential of 76% neutrophils. C-reactive protein was 125.6 mg/L (normal reference values, 0–8 mg/L). A cold agglutinin titer of *M pneumoniae* IgM was high at 1:2560. A high resolution CT scan of the chest revealed a consolidation in the right lower lobe and massive pleural effusion (Fig. 1b). The boy was given cefoperazonesulbactam (50 mg/kg, 3 times daily) and azithromycin (10 mg/kg, once daily). Fever still persisted at 72 hours post-admission. In order to exclude other febrile diseases, an echocardiography was carried out and surprisingly revealed a “mass” attached to the lateral wall of the left atrium extending into the right lower pulmonary vein (Fig. 2). A lab work-up was conducted for prothrombotic conditions. Coagulation studies revealed increased fibrin D-dimer level (3.38 mg/L; normal reference values, 0–0.55 mg/L) and fibrinogen (636 mg/dl; normal reference values, 170–450 mg/dl). Prothrombin time was in the normal range. Laboratory evaluations revealed that the patient tested strongly positive for anticardiolipin IgM but negative for anticardiolipin IgG antibody, lupus anticoagulant and antinuclear antibody. Protein C and S levels were within the normal range. We initiated the treatment with enteric coated aspirin (4 mg/kg body weight, once daily), low molecular weight heparin calcium (100IU/kg body weight, once daily) for standard anticoagulant therapy.

After 10 days of treatment, the childs body temperature returned to normal. Chest X-ray revealed that right lung shadows and pleural effusion decreased compared with before. But the thrombus in left atrium was almost the same size by echocardiography. D-dimer test was 3.32 mg/L.

**Figure 3:**
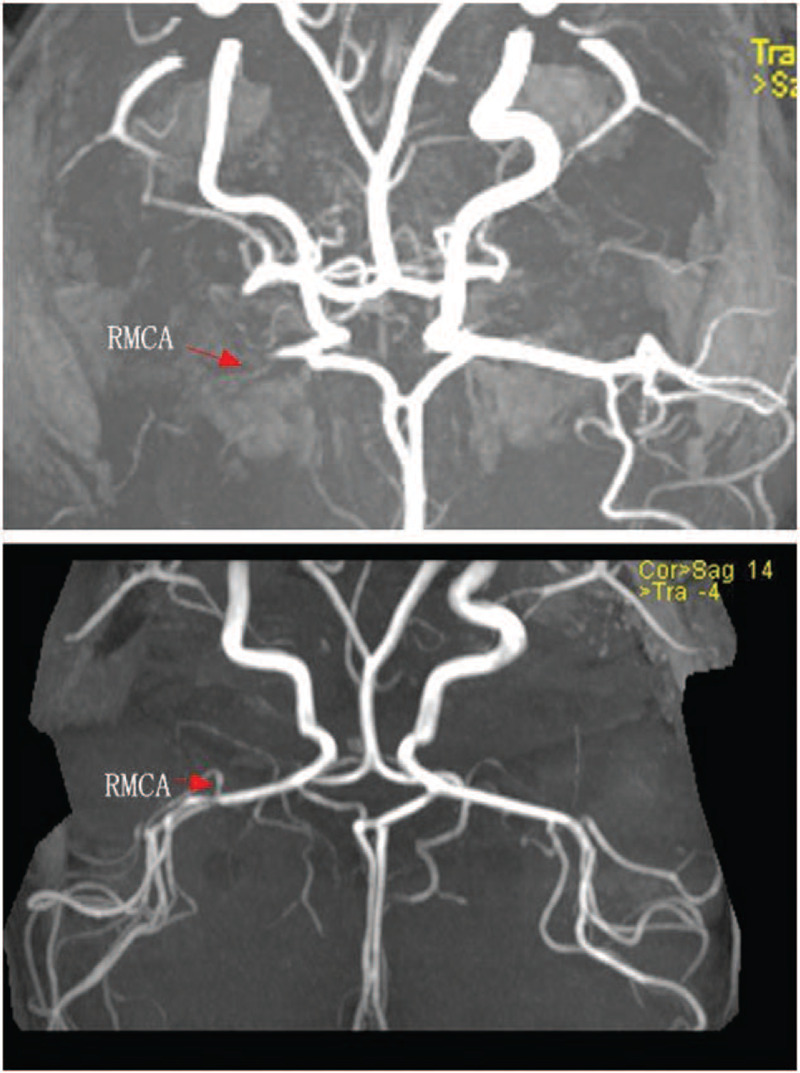
Magnetic resonance angiography (MRA). a. MRA failed to visualize the right cerebral artery and its distal branches. b. A repeat MRA revealed that right middle cerebral artery patency than before. RMCA = right middle cerebral artery.

At day 12 after admission, the patient showed impaired consciousness, aphasia, flattened right nasolabial fold, and right deviation of the protruded tongue after washing hand. The muscle strength of the left upper and lower limb was grade 1, and that of the right upper and lower limb was grade 3. Magnetic resonance angiography (MRA) failed to visualize the right cerebral artery and its distal branches (Fig. 3a), which implied the right middle cerebral artery infarction. Diffusion weighted imaging (DWI) demonstrated hyperintensity in the right frontoinsular cortex. Considering the possibility of stroke due to cardiac thrombosis, an emergency echocardiography was arranged which showed the thrombus in left atrium was still the same size. Urokinase thrombolytic therapy lasted 24 hours and continued with low molecular heparin calcium and aspirin. We continued to monitor the coagulation function of the child and found that the D-dimer decreased slowly with normal prothrombin time. Serial echocardiograms showed a steadily decreasing size of thrombus with eventual disappearance at day 22 after admission. The childs parents expressed concern about the effectiveness of the treatment, but actively cooperated with the examination and rehabilitation.

After 30 days of hospitalization, the patient was discharged and transferred to the rehabilitation department for further treatment, continuing with low molecular heparin calcium and aspirin. On follow up, echocardiography showed no new thrombosis in the left atrium at day 7 post-discharge. A repeat MRA revealed that right middle cerebral artery patency than before, with serious cerebral edema (Fig. 3b). After 5 weeks post-discharge, the fibrin D-dimer level decreased to 0.9 mg/L. His right upper and lower limb muscle power was normal and the left upper and lower limb muscle power was grade 3 after rehabilitation for 2 months. Six months after discharge, the child was followed-up in outpatient department and thrombogenesis recurrence were not reported during this period.

## 3 Discussion

*Mycoplasma pneumoniae* is a common cause of community-acquired pneumonia in school-aged children and adolescents. *M pneumoniae* infection has been recognized to be associated with many extra-pulmonary manifestations, including neurologic, cardiac, dermatologic, hematologic, immunological and gastrointestinal complications, which pathomechanisms remain largely unknown.^[1]^

It has been reported that children with severe *M pneumoniae* pneumonia had a high risk of blood coagulation and thrombosis.^[2]^ Liu *et al*^[3]^ reported a series of 43 pediatric *M pneumoniae* pneumonia associated thromboses cases with diverse clinical manifestations. They found that pulmonary consolidation (>2/3 lobe) and a high level of inflammatory markers (CRP >97.5 mg/L and LDH >735.1 IU/L) were risk factors that were strongly associated with thromboses.

There are currently only a few cases of mycoplasma infection with intracardiac thrombosis reported in the literature.^[3,4–6]^ Intracardiac thrombosis can be the only type of thrombosis present in a patient, and it can be asymptomatic. It often occurs in the right heart chamber and close to the tricuspid valve. In our case, thrombosis of the left atrium was found accidentally by echocardiography as the first manifestation. If the thrombus in left atrial falls off, it can cause multiple arterial thromboses, which is life-threatening.

The mechanisms underlying *M pneumoniae* infection-associated thrombosis remain unclear. One suggested possible mechanism is that antiphospholipid antibodies induced by *M pneumoniae* infection result in a transient hypercoagulable state, because positive ANA and aCL-IgM were found in more than 50% of patients with *M pneumoniae* infection, especially *M pneumoniae* -associated thrombosis.^[3,7]^ These findings suggest that the autoimmune inflammation caused by antiphospholipid antibodies plays a crucial role in the process of thrombosis. These antibodies in most cases disappear during convalescence and the hypercoagulable state does not last for many months.^[1]^

There is no uniform standard for the treatment of mycoplasma pneumonia complicated with intracardiac thrombus. The main treatments include anticoagulation therapy, thrombolytic therapy and surgical thrombectomy.^[8]^ The choice of treatment depends on the patients clinical parameters. Based on available clinical literature, we believe that anticoagulant therapy for small intracardiac thrombosis is an effective method and relatively safe with low molecular weight heparin.

In the treatment of this case, the lung condition of the child improved after strong antibiotics and anticoagulation therapy. But the D-dimerlevel remained high for a long time. D-dimer is a specific degradation product of cross-linked fibrin, which has high value for the diagnosis, observation of curative effect and prognosis of blood hypercoagulable state and thrombotic diseases.^[9,10]^ Liu et al^[3]^ suggested that D-dimer >5.0 mg/L, particularly >11.1 mg/L would help the early diagnosis of thrombosis. The continued high level of D-dimer indicated that the causes of the hypercoagulable state should be considered.

The cause of stroke and obstruction of the right middle cerebral artery during treatment is still unclear. It has been speculated that *M pneumoniae* may disrupt the integrity of the vascular endothelium and upset the equilibrium between coagulation and anticoagulation by eliciting an inflammatory response, which may lead to hypercoagulation and thrombosis.^[11,12]^ A direct invasion mechanism has been proposed in patients with stroke because *M pneumoniae* DNA was detected in cerebrospinal fluid.^[13]^ Gu et al^[14]^ reported a series of pediatric cases of *M pneumoniae* pneumonia, including 1 case complicated with occlusion of the right MCA and cerebral infarction and 1 case with occlusion of the basilar artery and posterior cerebral artery. Thrombolytic therapy is considered the optimal modality for acute ischemic stroke in adults; however, the optimal approach for managing pediatric stroke remains elusive. Gu et al^[14]^ treated 2 children with cerebral infarction with thrombolytic therapy; 1 died of brain herniation while the other lost consciousness. In our case, cerebral vascular obstruction caused by shedding of the thrombus in left atrium cannot be ruled out. So urokinase thrombolytic therapy lasted 24 hours with low molecular heparin calcium and aspirin, with monitoring of the coagulation function.

There is evidence that aberrant host immune responses play a critical role in the development of extrapulmonary manifestations due to *M pneumoniae* infections. Therefore, immunomodulators, such as corticosteroids or immunoglobulins, may be beneficial for the severe cases.^[15]^ For the patients with vascular occlusion manifestations, anticoagulation therapy should be used as soon as possible.^[16]^

In conclusion, for the patients with high risk factors for embolism, such as obvious systemic inflammatory response and significantly increased D-dimer, we should pay more attention to thrombotic symptoms. D-dimer level and anti-phospholipid antibodies should be routinely monitored. Anticoagulation therapy should be used as soon as possible for the patients with vascular occlusion manifestations. *M pneumoniae* pneumonia complicated with cardiac thrombus and cerebral infarction in children is very rare, and early diagnosis and treatment with multiple modalities maybe useful for improving prognosis.

## Author contributions

**Conceptualization:** Yefeng Wang, Ningan Xu.

**Data curation:** Yefeng Wang.

**Investigation:** Yefeng Wang.

**Methodology:** Xicheng Deng, Ningan Xu.

**Supervision:** Zhi Chen.

**Writing – original draft:** Yefeng Wang.

**Writing – review & editing:** Yunbin Xiao, Xicheng Deng.

## References

[1] Narita M Classification of extrapulmonary manifestations due to mycoplasma pneumoniae infection on the basis of possible pathogenesis. Front Microbiol 2016;7:23. 26858701 10.3389/fmicb.2016.00023 PMC4729911

[2] Fumarola D Intravascular coagulation and Mycoplasma pneumoniae infection. Pediatr Infect Dis J 1997;16:1012–3. 9380463 10.1097/00006454-199710000-00029

[3] Jinrong Liu Ruxuan He Runhui Wu Mycoplasma pneumoniae pneumonia associated thrombosis at Beijing Children's hospital. BMC Infect Dis 2020;20:51. 31948402 10.1186/s12879-020-4774-9 PMC6966865

[4] Bakshi M Khemani C Vishwanathan V Mycoplasma pneumonia with antiphospholipid antibodies and a cardiac thrombus. Lupus 2006;15:105–6. 16539282 10.1191/0961203306lu2258cr

[5] Nagashima M Higaki T Satoh H Cardiac thrombus associated with Mycoplasma pneumoniae infection. Interact CardiovascThorac Surg 2010;11:849–51. 10.1510/icvts.2010.242115 20847069

[6] Li QR Yuan Y Lin L Mycoplasma pneumoniae pneumonia complicated with cardiac thrombosis in children: report of 2 cases. Chin J Pediatr 2018;56:950–1. 10.3760/cma.j.issn.0578-1310.2018.12.012 30518011

[7] Char M Witmer Andrew P Steenhoff Samir S Shah Mycoplasma pneumoniae, splenic infarct, and transient antiphospholipid antibodies: a new association? Pediatrics 2007;119:e292–5. doi:10.1542/peds.2006-1340 PMID: 17178923. 17178923 10.1542/peds.2006-1340

[8] Egolum UO Stover DG Anthony R Intracardiac thrombus: diagnosis, complications and management. Am J Med Sci 2013;345:391–5. 23328835 10.1097/MAJ.0b013e318272b0b0

[9] Kanis J Pike J Hall CL Clinical characteristics of children evaluated for suspected pulmonary embolism with D-dimer testing. Arch Dis Child 2018;103:835–40. 29117964 10.1136/archdischild-2017-313317

[10] Hennelly KE Baskin MN Monuteuax MC Detection of pulmonary embolism in high-risk children. J Pediatr 2016;178:214–8. 27567411 10.1016/j.jpeds.2016.07.046

[11] Jin X Zou Y Zhai J Refractory Mycoplasma pneumoniae pneumonia with concomitant acute cerebral infarction in a child: a case report and literature review. Medicine (Baltimore) 2018;97:e0103. 29595632 10.1097/MD.0000000000010103 PMC5895422

[12] Sarathchandran P Al Madani A Alboudi AM Mycoplasma pneumoniae infection presenting as stroke and meningoencephalitis with aortic and subclavian aneurysms without pulmonary involvement. BMJ Case Rep 2018;2018. pii: bcr-2017-221831. doi: 10.1136/bcr-2017-221831.PMID: 29326371. 10.1136/bcr-2017-221831 PMC5778324 29326371

[13] Padovan CS Pfister HW Bense S Detection of *Mycoplasma pneumoniae* DNA in cerebrospinal fluid of a patient with *M. pneumoniae* infection-"associated" stroke. Clin Infect Dis 2001;33:E119–21. 11595996 10.1086/323461

[14] Gu HY Zhao DY Wang Q Clinical analysis of 6 cases of Mycoplasma pneumoniae pneumonia complicated with embolism. Chin J Appl Clin Pediatrics 2016;31:288–91.

[15] Spuesens EB Meyer Sauteur PM Vink C Mycoplasma Pneumoniae Infections--Does Treatment Help? J Infect 2014;69: Suppl 1: S42–6. 25267596 10.1016/j.jinf.2014.07.017

[16] Chen Y Huang P Chen Q Two separated thrombi in deep veins associated with pulmonary embolism after mycoplasma pneumoniae infection: a case in adolescent female. Transl Pediatr 2013;2:198–201. 26835314 10.3978/j.issn.2224-4336.2013.10.01 PMC4729075

